# Artificial intelligence and predictive models for early detection of acute kidney injury: transforming clinical practice

**DOI:** 10.1186/s12882-024-03793-7

**Published:** 2024-10-16

**Authors:** Tu T. Tran, Giae Yun, Sejoong Kim

**Affiliations:** 1 Department of Internal Medicine, Thai Nguyen University of Medicine and Pharmacy, Thai Nguyen, Vietnam; 2 Department of Nephro-Urology and Dialysis, Thai Nguyen National Hospital, Thai Nguyen, Vietnam; 3 Department of Internal Medicine, Seoul National University College of Medicine, Seoul, Republic of Korea; 4 Department of Internal Medicine, Hallym University Dongtan Sacred Heart Hospital, Hwaseong, Republic of Korea; 5 Department of Internal Medicine, Seoul National University Bundang Hospital, Seongnam, Republic of Korea; 6 Center for Artificial Intelligence in Healthcare, Seoul National University Bundang Hospital, Seongnam, Republic of Korea

**Keywords:** Acute kidney injury, Early detection, Prediction models, Artificial intelligence, Machine learning

## Abstract

Acute kidney injury (AKI) presents a significant clinical challenge due to its rapid progression to kidney failure, resulting in serious complications such as electrolyte imbalances, fluid overload, and the potential need for renal replacement therapy. Early detection and prediction of AKI can improve patient outcomes through timely interventions. This review was conducted as a narrative literature review, aiming to explore state-of-the-art models for early detection and prediction of AKI. We conducted a comprehensive review of findings from various studies, highlighting their strengths, limitations, and practical considerations for implementation in healthcare settings. We highlight the potential benefits and challenges of their integration into routine clinical care and emphasize the importance of establishing robust early-detection systems before the introduction of artificial intelligence (AI)-assisted prediction models. Advances in AI for AKI detection and prediction are examined, addressing their clinical applicability, challenges, and opportunities for routine implementation.

## Background

Acute kidney injury (AKI) is a common and serious clinical condition characterized by a rapid decline in kidney function, often resulting from factors such as sepsis, hypovolemia, and nephrotoxic medications [1, 2]. AKI is associated with increased morbidity, mortality, and healthcare costs, making its early detection and prediction crucial for improving patient outcomes [2–5]. The prevalence of AKI in hospitalized patients ranges from 6 to 18%, and its incidence tends to increase during hospitalization [6, 7]. In Korea, the National Health Insurance Review and Assessment database revealed that the incidence of AKI increased from 7.4% in 2008 to 8.3% in 2015 [8]. This trend is also evident in pediatric populations, with AKI affecting approximately 26% of hospitalized children [9] and up to 29.9% of critically ill neonates [10]. The impact of AKI extends beyond clinical outcomes—patients with AKI reportedly have higher in-hospital mortality rates, longer intensive care unit (ICU) stays, and greater total costs [8]. For example, a nationwide study in France revealed that patients with AKI had significantly higher hospital expenses and longer stays than patients without [11]. According to the Pediatric Health Information System, the adjusted charges per AKI case from 2019 to 2021 were $72,460 [5].

Traditional methods for AKI detection rely on clinical markers, such as serum creatinine (SCr) levels and urine output, which often only reveal kidney damage after it has occurred. The delay in diagnosis can lead to suboptimal patient management and poorer outcomes. Additionally, these markers can be influenced by various non-kidney-related factors, leading to diagnostic inaccuracies. Tools with a higher predictive value and precision that can be used to identify AKI at an earlier stage are urgently needed, allowing for interventions to prevent progression to more severe injury. Artificial intelligence (AI) has emerged as a transformative tool, offering potential solutions to these diagnostic challenges. Over the past decade, substantial efforts have been made to develop and validate predictive models for AKI by using clinical data, biomarkers, and advanced analytics techniques. These models aim to identify patients at high risk of AKI before the onset of overt kidney injury, enabling timely interventions to prevent or mitigate adverse effects.

With the recent advent of AI, more dynamic models for the early detection and prediction of AKI have been developed using machine learning (ML) algorithms. Various ML-based risk-stratification models have been evaluated in different clinical settings [12–14]. In this review, we examine the latest advancements, key studies, and future directions in AI for the early detection and prediction of AKI, focusing on clinical applicability, integration challenges, and opportunities.

## Current challenges in AKI detection and prediction

### AKI diagnosis

According to Kidney Disease: Improving Global Outcomes (KDIGO) criteria, AKI is defined according to changes in kidney function, including the SCr levels and urine output, within 48 h or 7 days [15]. However, as SCr levels and urine output can be affected by non-kidney-related factors, they are imperfect indicators of a reduced glomerular filtration rate (GFR) [16]. Therefore, the diagnosis of AKI based on KDIGO criteria should be considered within the clinical context. The prolonged half-life of creatinine and the existence of kidney functional reserve delay the accurate reflection of GFR and, therefore, the recognition of kidney dysfunction [17, 18]. Cystatin C, another marker of glomerular filtration, may be useful in the setting of muscle wasting but is also affected by comorbidities [19–21].

To improve AKI detection, diagnostic methods are being developed to improve AKI detection in four main categories: clinical risk scores, biomarkers, electronic alerts (e-alerts), and real-time prediction models. —Each of these approaches offers unique benefits—clinical risk scores are useful for identifying patients risk of developing AKI, while e-alerts are more valuable for early detection after the onset of AKI. Real-time prediction models offer the potential to monitor patients continuosly, providing predictive insights both before and after AKI develops [22].

### Clinical risk scores

Risk-prediction scores for AKI have been described in various clinical settings, including critical care, surgery, and contrast-induced nephropathy [23–25]. The Simple Postoperative AKI Risk (SPARK) index, used in non-cardiac surgeries, is a noteworthy example [26]. The SPARK index comprises a summation of integer scores assigned to the following variables: age, sex, expected surgery duration, emergency operation, diabetes mellitus, use of renin-angiotensin-aldosterone inhibitors, baseline estimated GFR (eGFR), dipstick albuminuria, hypoalbuminemia, anemia, and hyponatremia [26]. For instance, the Mehran risk score predicts AKI risk after percutaneous coronary intervention and has exhibited adequate performance upon external validation [27]. Large cohort studies have revealed that surgery, particularly cardiac surgery, is a major cause of AKI. Risk-scoring systems, such as the European Heart Surgery Risk Assessment System; age, creatinine, ejection fraction score, and Society of Thoracic Surgeons score, have been developed to predict AKI risk after cardiac surgery [28–30]. Despite their utility, these scores often lack external validation and personalization.

Accurate risk-prediction scores should guide clinicians in the identification of at-risk patients, conducting of additional diagnostic tests, and suggestion of preventive or therapeutic interventions. Such models can be developed and validated according to guidelines provided by the transparent reporting of a multivariable prediction model for individual prognosis or diagnosis (TRIPOD) initiative [31].

### AKI biomarkers

**Fig. 1:**
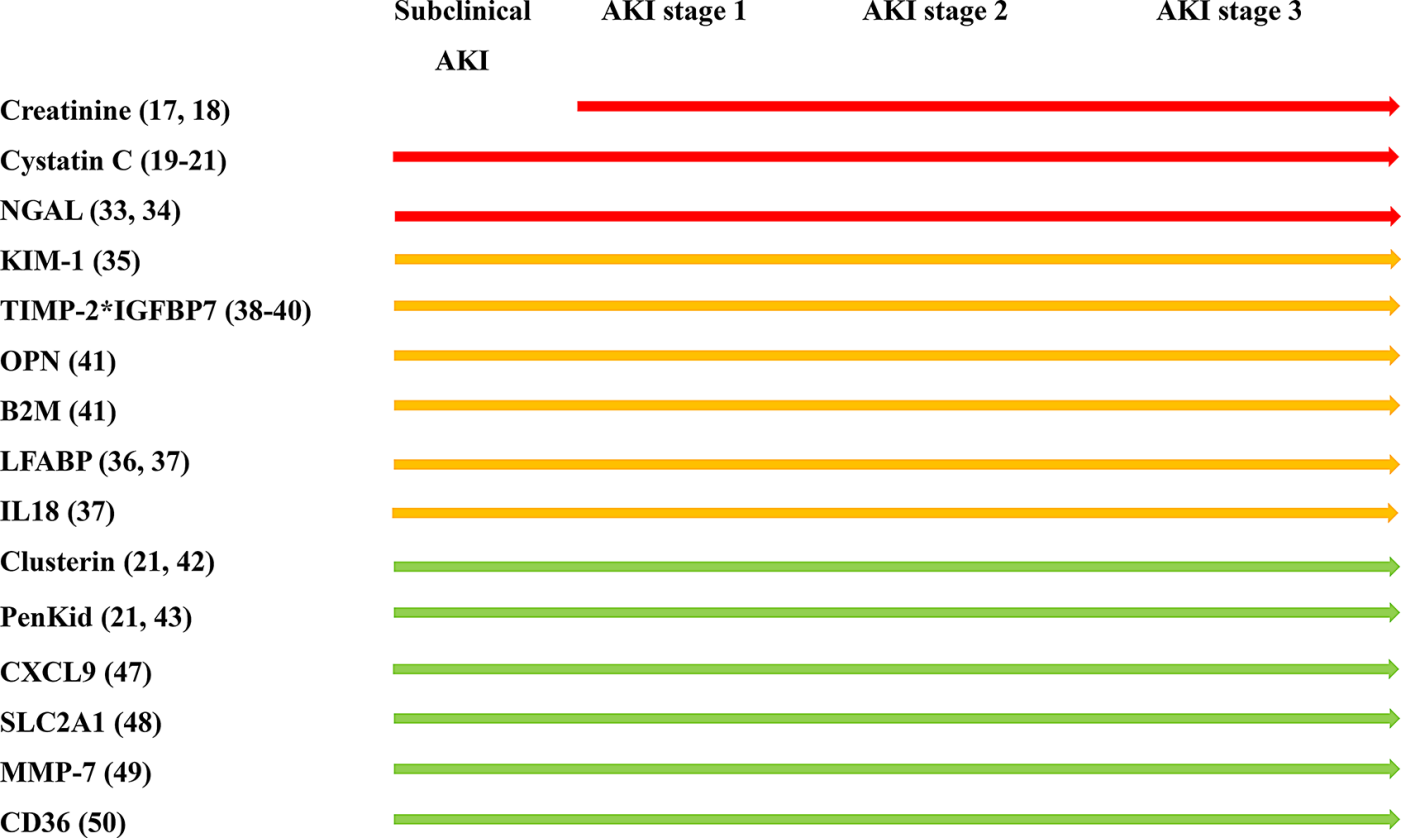
**Summary of AKI biomarkers**. Abbreviations: AKI, acute kidney injury; NGAL, neutrophil gelatinase-associated lipocalin; KIM-1, kidney injury molecule-1; TIMP-2*IGFBP7, tissue metalloproteinase-2 and insulin-like growth factor binding protein-7; OPN, osteopontin; B2M, β-2 microglobulin; LFABP, liver fatty acid-binding protein; IL 18, interleukin-18; penKid, proenkephalin A 119–159; CXCL9, chemokine C-X-C motif ligand 9; SLC2A1, solute carrier family 2 member 1; MMP-7, matrix metalloproteinase 7; CD36, cluster of differentiation 36. **Red**: Strong evidence. **Yellow**: Medium evidence. **Green**: Weak evidence.

To address the issue of delayed AKI diagnosis when relying solely on SCr levels, Cystatin-C levels, and urine output, new biomarkers have been identified that can indicate kidney injury before it meets the KDIGO criteria for AKI, known as “subclinical AKI” [32] (Fig. 1). Early detection via biomarkers, such as neutrophil gelatinase-associated lipocalin (NGAL) [33, 34], kidney injury molecule 1 (KIM-1) [35], liver fatty acid-binding protein (LFABP) [36, 37], urinary tissue inhibitor of metalloproteinase 1 [38], and the combination of tissue inhibitor of metalloproteinase 2 and insulin-like growth factor-binding protein 7 (sold under the trade name NephroCheck) [39, 40], osteopontin, β-2 microglobulin [41], clusterin [42], penKid [43] can prompt timely diagnostic and preventive measures [44]. These biomarkers have proven effective in the identification of high-risk patients for clinical trials in which early-prevention strategies are investigated [45, 46].

Recent studies have made considerable advancements in the identification of novel diagnostic biomarkers and mechanisms involved in AKI, cluding its subtypes such as acute interstitial nephritis (AIN). For instance, urine proteomics and tissue transcriptomics have been used to identify chemokine (C-X-C motif) ligand 9 as a promising, noninvasive diagnostic biomarker for AIN [47]. Bioinformatics and ML methods have been used to screen for ferroptosis-related genes, solute carrier family 2 member 1(SLC2A1*)*, associated with AKI [48]. Further, an unbiased kidney proteomics and transcriptomics approach has been used to identify matrix metalloproteinase 7 as the protein most strongly associated with kidney fibrosis and eGFR, providing valuable information on tubular injury in focal segmental glomerulosclerosis and minimal change disease [49]. Recent results highlight a novel mechanism involving cluster of differentiation 36 in cisplatin-induced AKI, suggesting potential therapeutic targets [50]. Despite substantial research efforts, clear guidance on the clinical application of AKI biomarkers remains lacking. The limitations of the SCr level as an AKI marker and challenges in patients without baseline kidney function highlight the need for improved diagnostic tools. Furthermore, barriers such as cost, availability, and limited therapeutic options hinder the widespread clinical adoption of potential biomarkers. Demonstrating their utility and effectiveness in real-world settings requires a comparison of two strategies: one in which clinicians have access to biomarker results and another in which they do not.

### AKI alerts

e-Alerts have been suggested as solutions in the early diagnosis of AKI [51, 52]. These alert systems are used to identify AKI in both community and hospital settings according to KDIGO criteria [53] (Table 1). Based on prevalent KDIGO criteria, these alerts are particularly beneficial in less-monitored non-ICU settings when linked to an order set and/or action [54]. However, evidence for their benefit in the ICU is limited [55]. For example, in 2014, Seoul National University Bundang Hospital implemented an electronic medical record-based AKI alert system, leading to markedly positive outcomes such as a reduction in overlooked AKI events, increased early nephrologist consultations, and a reduction in severe AKI occurrences [7]. While this system showed promising results, studies on e-alerts have yielded mixed coutcomes. Some studies indicate improvements in mortality and length of stay, whereas others revealed no significant changes in clinical outcomes [53, 57]. Further studies were conducted to assess the effectiveness of AKI e-alerts as a part of care bundles. Such care bundles reportedly improve care behaviors and reduce severe AKI events, although they may not enhance kidney function or overall patient-centered outcomes [56, 58]. In community settings, AKI alerts facilitate earlier responses, allowing for more optimal management of community-acquired AKI, where patients may not be as closely monitored [59, 60]. Although e-alerts can increase early consultations with nephrologists and awareness of AKI, a 2017 systematic review concluded that e-alert systems do not significantly impact mortality or dialysis incidence [61]. Similarly, Wilson FP et al. concluded that alerts did not reduce the risk of the primary outcome for hospitalized patients with AKI and found that mortality was worse in non-teaching hospitals [62]. To further enhance AKI management, combining AI-driven early detection with predictive analytics offers a more proactive approach than traditional e-alerts, providing a critical window for preventive interventions toward the improvement of patient outcomes [63].

**Table 1:** Summary of AKI alerts

Type of Study	*N*	Duration	Algorithm	Main results
**Primary care**				
Barton et al., 2020 (53) Retrospective Observational Study	2742 (Pre-alert: 991) (Post-alert: 1751)	March 2017-February 2018	NHS England AKI-detection algorithm	• 1784 episodes of AKI identified over 12 months • Significant reduction in median length of stay and mortality rates for patients with AKI • Overall improvement in mortality and reduction in length of stay for patients with AKI3
Holmes et al., 2017 (60) Prospective national cohort study	21,093	November 2013-April 2016	Biochemistry-based AKI e-alert system; Welsh National Health Service	• 28.8% of CA-AKI e-alerts were PC-AKI • Lower 90-day mortality for PC-AKI than that for non-PC-AKI • Impact of repeat biochemistry tests on patient outcomes
Aiyegbusi et al., 2019 (61) Retrospective observational study	3462 (Pre-alert: 2257) (Post-alert: 1205)	January 2012-December 2012, April 2015-May 2016	NHS Tayside biochemistry laboratory reporting system	• Comparison of AKI e-alerts and repeat blood testing durations • Increase in repeat biochemistry tests after implementation • Increase in hospitalization rates within 7 days of AKI after implementation
In hospital				
Al-Jaghbeer et al., 2018 (54) Retrospective cohort study	528,108	October 2012 – September 2015	Clinical decision support system (University of Pittsburgh Medical Center )	• AKI was diagnosed in 64,512 patients (12.2%). • The crude mortality rate for patients with AKI decreased from 10.2% before to 9.4% after CDSS implementation (odds ratio 0.91; *p* = 0.001), while mortality rates for patients without AKI remained unchanged • The mean hospital stays for patients with AKI decreased from 9.3 to 9.0 days (*p* < 0.001), with no change for those without AKI.
Colpaert K, et al., 2012 (55) Prospective intervention study.	951	January 1, 2007 - June 27, 2007	AKI sniffer	• In a study of 2593 acute kidney injury alerts, most alerts were classified as RIFLE class risk (59.8%). • Patients in the alert group received therapeutic interventions more quickly. • A higher percentage of alert group patients received fluid therapy (23.0% vs. 4.9% and 9.2%, *p* < 0.01), diuretics (4.2% vs. 2.6% and 0.8%, *p* < 0.001), and vasopressors (3.9% vs. 1.1% and 0.8%, *p* < 0.001)
Park et al., 2018 (56) Before-and-After quality improvement study	3193 (Usual-care group: 1884; Alert group: 1309)	January 2013-June 2015	AKI alert system (KCT0002010)	• Reduced overlooked AKI events • Increased early nephrologist consultation • Improved AKI recovery • No significant impact on mortality by the alert system
Niemantsverdriet et al., 2023 (58) Before and after study	Before implementation: 866 After implementation: 853	April 6, 2021-April 5, 2022	Laboratory Information System (GLIMS 9.9.6; Clinisys, Gent, Belgium).	• Improved AKI awareness and patient management after implementation
Chen-Xu et al., 2024 (59) Retrospective single-center cohort study	5728	September 2018-July 2021	AKI order set and electronic AKI care plan	• The utilization of AKI order sets resulted in enhancements in all-cause mortality and renal function, but also led to longer length of stay among patients with AKI at West Suffolk Hospital
Li et al., 2024 (57) Randomized Clinical Trial	2208	August 2019-December 2021	Electronic AKI alert	• The AKI alert did not enhance kidney function or other patient-centered outcomes but did impact patient care behavior

Abbreviations: AKI, acute kidney injury; AKI3, acute kidney injury stage 3; CA-AKI, community-acquired acute kidney injury; e-alert, electronic alert; NHS, National Health Service; PC-AKI, primary-care acute kidney injury; CDSS, clinical decision support system.

We have selected recent research from PubMed that represents a diverse range of settings, including community and hospital environments such as ICU and non-ICU units, as well as studies from various regions, including Europe, Asia, and others.

## AI and ML in AKI

### Overview of ML algorithms

In the context of AKI, AI refers to the use of machines designed to replicate human cognitive functions, such as learning and problem-solving, to enhance and early detection and treatment. ML, a subset of AI, involves algorithms that can learn from clinical data and predict AKI outcomes without explicit programming, offering new possibilities in kidney disease management. The ML process can be categorized into supervised, unsupervised, semi-supervised, and reinforcement learning, depending on the type of data and the specific task at hand. In supervised machine learning, a computer is trained to predict a labeled output based on one or more inputs, known as features. Common supervised learning algorithms in AKI prediction include linear regression, logistic regression (LR), random forests (RF), gradient boosting machines (GBM), extreme gradient boosting (XGBoost), and deep neural networks. More advanced techniques, such as ridge regression, LASSO, and elastic net, are used to create parsimonious models that avoid overfitting, thereby enhancing generalizability. In unsupervised machine learning, on the other hand, there are one or more inputs but no labeled outputs, allowing models to conver hidden patterns in the data. This approach helps identify the structure of the data, identifying patterns and groupings, or clusters within it [64].

ML algorithms have become increasingly prominent in the early detection and management of AKI [12]. Key ML algorithms used in AKI prediction include LR, k-nearest neighbors, support vector machines, decision trees, RF, XGBoost and artificial neural networks [65]. These algorithms analyze large datasets from electronic health records (EHRs) to identify patterns and predict the onset of AKI with high accuracy [65, 66]​. One of the most popular ML techniques, logistic regression, uses a sigmoid function to predict the connection between a dependent binary variable and one or more independent variables. However, it is limited in terms of continuous outcomes [66]. Deep learning (DL), a subset of ML, mimics the human brain’s inner layers, creating knowledge from multiple layers of information processing. DL capabilities improve as the size of the dataset increases, rendering it highly effective in AKI-prediction models​ [12]. These concepts form a hierarchy: AI includes ML, and ML includes DL, with each playing a crucial role in advancing intelligent systems and applications.

### Specific AI models and their applications

AI models have been developed to predict AKI in various clinical settings (Table 2) [67–70]. For example, models based on Logistic regression, gradient boosting, and eXtreme Gradient Boosting have demonstrated high predictive power in ICU settings ​​ [65, 71, 72]. These models are commoly trained using large datasets like the Medical Information Mart for Intensive Care III and eICU Collaborative Research databases. However, challenges such as incomplete data and limited generalizability across diverse patient populations remain [66]​​. Additionally, models have been used to predict adverse outcomes of AKI and generate postoperative AKI-detection models [73–75]. For instance, during the coronavirus disease 2019 (COVID-19) pandemic, an AI model was utilized to create the predictive AKI-COV score based on coefficients from the Elastic Net model. This score assists healthcare workers in identifying hospitalized COVID-19 patients at risk for AKI, enabling more intensive monitoring and more effective resource allocation [68]. In community settings, logistic regression and recurrent neural network models have demonstrated their ability to assist both primary and secondary care physicians in identifying patients at high risk of AKI [76, 77]. Furthermore, ML has been applied to pediatric critical care patients, accurately predicting moderate to severe AKI up to 48 h before its onset. This early detection allows for timely interventions, such as adjusting medications, potentially reducing the severity of AKI and improving patient outcomes [78].

**Table 2:** Summary of AI in AKI-prediction models

Type of Study	*N*	Duration	Setting	Algorithm	Results
**Primary Care**					
Bell et al., 2020 (78) Retrospective cohort study	273,450	January 2004-December 2012	Tayside region of Scotland; Kent, UK; Alberta, Canada	LR	A risk score with four variables showed good predictive performance, with C-statistics ranging from 0.71 to 0.80
Tomašev et al., 2019 (79) Retrospective cohort study	703,782	October 2011-September 2015	172 inpatient and 1062 outpatient sites, US Department of Veterans Affairs, USA	RNNs	The model accurately predicts 55.8% of all inpatient episodes of AKI and 90.2% of cases requiring subsequent dialysis, with a lead time of up to 48 h and a false alert-to-true alert ratio of 2:1
**In Hospital**					
Koyner et al., 2018 (72) Observational cohort study	121,158	November 2008-January 2016	ICU, wards, and emergency department; tertiary, urban, academic medical center, USA	GBM	The AUC was 0.90 to predict AKI2 within 24 h and 0.87 within 48 h The AUC was 0.96 to predict the need for kidney replacement therapy (*n* = 821) in the next 48 h
Ponce et al., 2021 (69) The cross-sectional multicenter prospective cohort study	870	May 2020-December 2020	Latin America AKI COVID-19 Registry	XGBoost, RF, and Elastic Net	The AKI-COV score had an AUC of 0.823 in the validation cohort
Zhang et al., 2021 (68) Retrospective cohort study	894	January 2015-September 2019	Liver transplantation, Third Affiliated Hospital of Sun Yat-sen University-Lingnan Hospital, China	LR, SVM, RF, and GBM	The GBM model achieved the highest AUC (0.76) to predict post-liver transplantation AKI
Dong et al., 2021 (80) Multicenter retrospective cohort study	16,863	2003–2019	PICU and cardiothoracic intensive care units of three independent tertiary-care pediatric intensive care centers; UK and US hospitals.	Multivariate Prediction Model	The model successfully predicted Stage 2/3 AKI before conventional criteria. The AUROC was 0.89. The ratio of false to true alerts for any AKI episodes was approximately one-to-one, resulting in a PPV of 47%. Among patients predicted to develop AKI, 79% received potentially nephrotoxic medication before the onset of AKI.
Thongprayoon et al., 2022 (75) single-center observational study	13,158	January 2014-December 2020	Cardiac surgery, Mayo Clinic Hospital, Rochester, MN	DT, RF, XGBoost, ANN, and LR	AutoML had the highest AUC (0.79), followed by RF, XGBoost, LR, ANN, and DT
Yue et al., 2022 (66) retrospective observational study	3176	June 2001-October 2012	ICUs, Beth Israel Deaconess Medical Center, Boston, MA	LR, KNN, SVM, DT, RF, XGBoost, and ANN	The XGBoost model achieved the highest predictive performance with an AUC of 0.821 to predict AKI in patients with sepsis
Schwager et al., 2023 (73) retrospective single-center cohort	89,429 Any-AKI cohort: 50,342 Moderate-to-severe cohort: 39,087	January 2005-December 2017	ICU, Mayo Clinic Hospital, Rochester, MN	LR	Models achieved an AUC of 0.756 at 6 h and 0.721 at 12 h before AKI diagnosis
Shin et al., 2023 (76) retrospective cohort study	785 Training set: 706 Test set: 79	May 2006-May 2019	Open or robotic partial nephrectomy at six tertiary referral centers in Korea.	XGBoost and genetic algorithms	The model-predicted immediate postoperative SCr levels correlated closely with the measured values (R^2^ = 0.9669) The sensitivity and specificity of the AKI-prediction model were 85.5% and 99.7% in the training set, and 100.0% and 100.0% in the test set, respectively
Kong et al., 2023 (77) retrospective cohort study.	134	January 2002-January 2022	Cardiopulmonary bypass, China	XGBoost, LR, LGBM, GNB, MLP, and SVM	The LR model had the best performance with a mean AUC of 0.89 in training sets and 0.84 in testing sets

Abbreviations: AI, artificial intelligence; AKI, acute kidney injury; AKI2, acute kidney injury stage 2; ANN, artificial neural network; AUC, area under the receiver operating characteristic curve; autoML, automatic machine learning; COV, coronavirus disease 2019; COVID-19, coronavirus disease 2019; DT, decision tree; eGFR, estimated glomerular filtration rate; GBM, gradient boosting machine; GNB, Gaussian Naive Bayes; ICU, intensive care unit; KNN, k-nearest neighbors; LGBM, Light Gradient-Boosting Machine; LR, logistic regression; MLP, multilayer perceptron; RF, random forest; RNN, recurrent neural network; SCr, serum creatinine; SVM, support vector machine; XGBoost, eXtreme Gradient Boosting; PICU, pediatric intensive care units; CTICU, cardiothoracic intensive care units; PPV, positive predictive value.

### Real-time prediction models

Real-time prediction models aim to provide immediate risk assessments by continuously analyzing patient data [79]. One example of the potential impact of real-time prediction models comes from a study by Koyner et al., which demonstrated that gradient-boosting models could predict AKI 24 to 48 h in advance with Area Under the Curve (AUC) values of 0.90 and 0.87, respectively [71]. These models do not rely solely on changes in creatinine levels, making them more robust to variations in baseline kidney function ​ [66, 79, 80].

### Comparative analysis of traditional vs. AI methods

Traditional AKI-prediction methods, such as logistic regression, have been widely used but often fall short in the handling of complex, high-dimensional data. In contrast, AI and ML models can manage large datasets and identify subtle patterns that traditional methods may miss. While ML models generally outperform traditional models in predictive accuracy, their complexity and and lack of interpretability can hinder clinical use​ [65, 81].

### Interpretability of AI models

While AI models, particularly complex ones such as deep learning algorithms, offer high predictive accuracy, they are often criticized for their ‘black box’ nature, which can hinder clinical decision-making. The lack of transparency in how predictions are generated may limit clinicians’ trust and the practical implementation of these models. To address this, various interpretability techniques, such as feature importance analysis and model-agnostic approaches, have been developed to provide insights into the decision-making process of AI models. By incorporating these techniques, AI-driven predictions can become more transparent and clinically usable, improving their acceptance and integration into healthcare practice.

## Application of AKI-prediction models in clinical practice

### Practical considerations for implementation

For AI-based prediction models to be effectively integrated into clinical practice, healthcare systems must address several practical barriers. These include data standardization across different healthcare systems, model validation to ensure applicability in diverse patient populations, and clinician training to interpret and act on AI-generated predictions [82]. Additionally, cost implications, data-integration, and clinician acceptance are notable hurdles that need to be addressed [83]. Collaborative efforts between AI specialists and healthcare providers are essential to ensure that predictive insights are actionable and reliable.

In addition to data and technical challenges, the complexity of AI models raises concerns about how well clinicians can understand and trust their predictions. This issue is especially important in critical care settings, where AI-based decisions can significantly affect patient outcomes. These challenges can be overcome by applying interpretability techniques that offer clear and understandable insights into AI models’ predictions. Enhancing transparency will be crucial for successfully integrating AI models into routine clinical practice.

### Case studies and real-world examples

Several important roles of AI in nephrology are emerging. Current applications include using ML algorithms to enhance the detection of urinary tract infections, employing image analysis for pathological evaluation in kidney transplantation, and predicting the progression of chronic kidney disease [84–86]. AI has the potential to mitigate the morbidity, mortality, and economic burden associated with hospital-based AKI by optimizing resource allocation, monitoring, and care delivery [87]. Newer AI models incorporate both baseline (pre-AKI) and dynamic, real-time data collected during hospital admissions, leading to improved predictive abilities [88].

Static AKI-risk models have been developed primarily in perioperative settings, notably in cardiac and cardiothoracic procedures [89]. For instance, Mangano et al. assessed postoperative kidney dysfunction and failure and identified preoperative predictors of kidney dysfunction, which were incorporated into prognostic scoring systems such as the “AKI following cardiac surgery” score [90, 91]. Amidst the COVID-19 pandemic, Ponce et al. used an ML approach to develop a prognostic score to predict in-hospital mortality in patients with AKI who also had COVID-19 [68]. Similarly, Thongprayoon et al. developed an automated ML model to predict cardiac surgery-associated AKI [73]. Such AI models have shown promise in the improvement of AKI prediction and management in various clinical settings.

### Overcoming challenges in clinical application

Despite advancements in AKI-prediction modeling, their translation into routine clinical practice remains limited. Challenges such as model complexity, data availability, and variability in patient populations pose significant barriers to their implementation. Furthermore, the lack of standardized protocols for AKI management and the need for real-time risk-assessment tools also hinder the adoption of prediction models in clinical decision-making. However, recent studies have demonstrated the potential clinical utility of AKI-prediction models in various healthcare settings, including ICUs, emergency departments, and surgical wards [68, 72, 74, 76]. By identifying high-risk patients at an early stage, these models have the potential to reduce AKI-related complications and improve patient outcomes via the timely initiation of preventive measures.

**Fig. 2:**
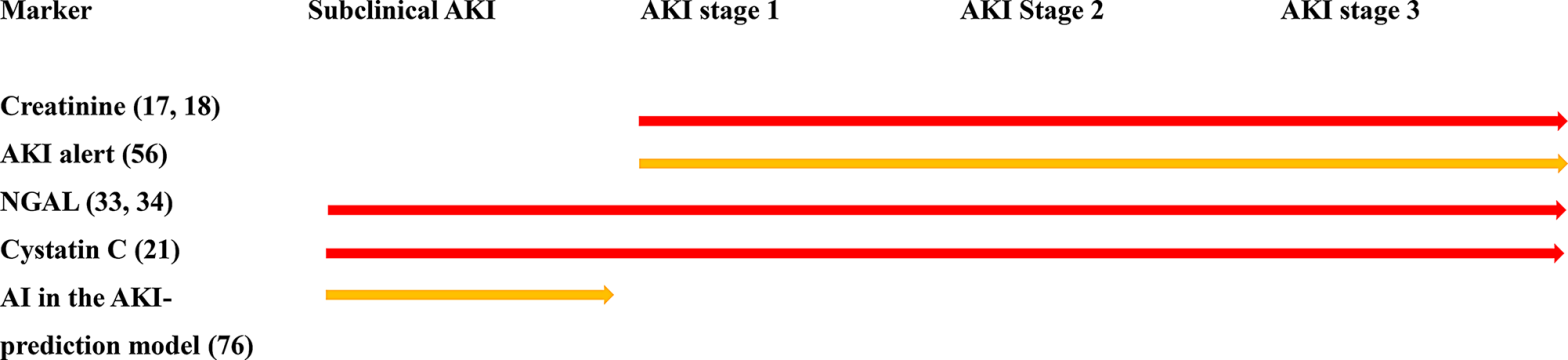
Summary of AKI diagnostic markers. Abbreviations: AKI, acute kidney injury; NGAL, neutrophil gelatinase-associated lipocalin; AI, artificial intelligence. **Red**: Strong evidence. **Yellow**: Medium evidence.

AI-based prediction models can play a dual role in addressing these challenges. On one hand, AI-based prediction models hold the potential to discover biomarkers for subclinical AKI, aligning with biomarkers such as NGAL and cystatin C, improving early detection. On the other hand, AKI-alert systems focus on predicting the clinical stage of AKI, based on traditional markers like SCr levels (Fig. 2). Both approaches, when integrated into clinical workflows, can contribute to earlier intervention and improved patient outcomes.

## Future directions

### Emerging trends in AI and AKI research

Future research should be focused on the refining of AI algorithms for better accuracy and the integration of multimodal data sources. The incorporation of dynamic, real-time data collected during hospital admissions may substantially enhance the predictive abilities of models. Studies have indicated that newer AI models, which incorporate both baseline and dynamic data, yield better predictive outcomes than static models. For instance, Tomašev et al.. utilized DL models to predict AKI 48 h in advance, demonstrating that the integration of time-series data from EHRs substantially improves predictive performance [77].

### Ethical considerations and patient privacy

The integration of AI in healthcare raises ethical and privacy concerns. Ensuring patient data privacy and addressing potential biases in AI models are critical to maintaining trust and confidence in the healthcare system. AI models can inadvertently perpetuate existing healthcare disparities if not carefully designed and validated [92–94]. Transparent reporting and adherence to ethical guidelines, such as those provided by the TRIPOD Initiative, are essential to foster trust in and promote the integrity of AI applications [31]. For example, in the development of AI models for AKI prediction, care is needed to ensure that these models do not disproportionately affect vulnerable populations. The implementation of robust data-anonymization techniques and regular audits of AI systems for bias can mitigate these risks.

### Areas for future research and development

Large-scale, multicenter studies are crucial to validate the clinical utility of AI-based AKI prediction models across diverse patient populations. Such studies should have the aim of generating generalizable results and ensure model robustness. Additionally, future research should be conducted to explore the development of personalized guidelines to optimize multidisciplinary-care plans, integrating both pre- and postoperative interventions and dynamic, real-time data collected during hospital admissions. Incorporating new biomarkers such as NGAL, KIM-1, and LFABP may enable timely diagnostic and preventive measures, especially for high-risk patients [33], reducing the progression to severe AKI and improving overall patient outcomes. Additionally, the potential of AI to monitor and manage other nephrology-related conditions, such as chronic kidney disease and urinary tract infections, should be further explored. Moreover, the integration of AI with telemedicine platforms may improve access to nephrology care, especially in underserved areas [95, 96]. For instance, AI-driven diagnostic tools can assist primary-care providers in remote settings by flagging high-risk patients for specialist referral, thereby improving early detection and treatment outcomes [97].

Additionally, while our review primarily focuses on the predictive accuracy and implementation of AI models for AKI detection, it is essential to consider the challenges associated with post-prediction actions and alert fatigue [62]. The effectiveness of AI models depends not only on accurate predictions but also on how these predictions are acted upon by healthcare providers. Frequent alerts may lead to alert fatigue, diminishing clinicians’ responsiveness to critical warnings. Future research should focus on strategies to reduce alert fatigue and enhance the effectiveness of post-prediction interventions, ultimately ensuring better patient outcomes.

## Conclusion

Moving forward, efforts should focus on enhancing the accuracy and generalizability of AKI-prediction models through large-scale, multicenter studies and external validation. Integrating predictive analytics into EHR systems may facilitate real-time risk assessment and decision-making support for clinicians. Standardization of AKI definitions and outcome measures is essential for the comparison of model performance across studies and populations. Moreover, education of patients and care providers on the importance of AKI prevention and early intervention is crucial to maximize the impact of prediction models in clinical practice. Alongside the technical challenges of implementing AI-based AKI prediction models, addressing the educational needs of healthcare providers is equally important. Proper education and training are essential for clinicians to effectively interpret and act on AKI alerts and predictions. Standardizing AKI responses across different hospitals and healthcare systems is another significant challenge that must be tackled to ensure consistent and effective care. Overcoming these challenges is crucial for maximizing the clinical impact of AI-driven prediction models and improving patient outcomes.

AKI early-detection and prediction models hold promise to improve patient care, but further research and implementation strategies are needed to realize their full potential in routine clinical practice. Although early detection remains crucial, the integration of AI-based prediction models represents the next frontier in AKI management. By combining these approaches, healthcare providers will be able to prevent and mitigate AKI with greater effectiveness, improving patient outcomes.

## Data Availability

No datasets were generated or analysed during the current study.

## Abbreviations

AI: artificial intelligence
AIN: acute interstitial nephritis
DL: deep learning
EHRs: electronic health records
e-alerts: electronic alerts
GBM: gradient boosting machines
GBM: gradient boosting machines
GFR: glomerular filtration rate
ICU: intensive care unit
KIM-1: kidney injury molecule 1
KDIGO: Kidney Disease: Improving Global Outcomes
LFABP: liver fatty acid-binding protein
LR: logistic regression
ML: machine learning
NGAL: neutrophil gelatinase-associated lipocalin
RF: random forests
SCr: serum creatinine
SPARK: Simple Postoperative AKI Risk
TRIPOD: transparent reporting of a multivariable prediction model for individual prognosis or diagnosis
XGBoost: extreme gradient boosting
